# Breastfeeding Insufficiencies: Common and Preventable Harm to Neonates

**DOI:** 10.7759/cureus.18478

**Published:** 2021-10-04

**Authors:** Vera K Wilde

**Affiliations:** 1 Methods, Ethics, and Technology, Independent Researcher, Berlin, DEU

**Keywords:** breastfeeding, autism spectrum disorder (asd), neonatal nutrition, starvation, neonatal jaundice, hypoglycemia, hyperbilirubinemia, hypernatremic dehydration, neurodevelopment, preventive health

## Abstract

Insufficient milk intake in breastfed neonates is common, frequently missed, and causes preventable hospitalizations for jaundice/hyperbilirubinemia, hypernatremia/dehydration, and hypoglycemia - accounting for most U.S. neonatal readmissions. These and other consequences of neonatal starvation and deprivation may substantially contribute to fully preventable morbidity and mortality in previously healthy neonates worldwide. Previous advanced civilizations recognized this problem of breastfeeding insufficiencies and had an infrastructure to solve it: Wetnursing, shared nursing, and prelacteal feeding traditions used to be well-organized and widespread.

Modern societies accidentally destroyed that infrastructure. Then, modern reformers missing a few generations of direct knowledge transmission about safe breastfeeding invented a new, historically anomalous conception of breastfeeding defined in terms of exclusivity. As that new intervention has become increasingly widespread, so too have researchers widely reported associated possible harms of the longer neonatal starvation/deprivation and later infant under-nutrition periods that it creates when breastfeeding is insufficient. Early insufficient nutrition/hydration has possible long-term effects including neurodevelopmental consequences such as attention deficit hyperactivity disorder, autism, cerebral palsy, cognitive and developmental delay, epilepsy, hearing impairment, kernicterus, language disorder, mood disorders, lower IQ, and specific learning disorder.

Current early infant feeding guidelines conflict with the available evidence. Recent reform efforts have tended to focus on using more technology and measurement to harm fewer neonates instead of proposing the indicated paradigm shift in early infant feeding to prevent more harm. The scientific evidence is already sufficient to mandate application of the precautionary principle to feed neonates early, adequate, and often milk before mothers’ milk comes in and whenever signs of hunger persist, mitigating possible risks including death or disability. In most contexts, the formula is the best supplementary milk for infants at risk from breastfeeding insufficiencies. National-level reviews of scientific evidence, health policy, and research methods and ethics are needed to initiate the early infant feeding paradigm shift that the data already support. Policy experiments and related legislative initiatives might also contribute to the shift, as insurers might decline or be required by law to decline reimbursing hospitals for costs of this type of preventable hospitalization, which otherwise generates profit.

## Introduction and background

Exclusive breastfeeding carries serious and under-recognized risks. An extensive literature shows that early adverse event exposures are associated with neurodevelopmental problems, and a large body of evidence links neonatal starvation specifically with a range of such problems. In the U.S., insufficient milk intake associated with breastfeeding contributes to the majority of 80,000 U.S. neonatal readmissions annually [1,2], mostly for jaundice/hyperbilirubinemia hypernatremia, dehydration, and hypoglycemia. In Nigeria, acute bilirubin encephalopathy (ABE) - an acute illness caused by severe hyperbilirubinemia - causes 5%-14% of neonatal deaths [3-5]; a study of nine major hospitals in five cities found over 15% of infants treated for hyperbilirubinemia had mild to severe bilirubin encephalopathy (including 35 deaths) [6]. ABE sits at one pole of a continuum of harms potentially associated with neonatal jaundice that also includes attention deficit hyperactivity disorder [7, 8], autism [9-11], cerebral palsy [12- 14], cognitive and developmental delay and disorder [15], epilepsy [16,17], hearing impairment [18,19], kernicterus [20] - severe bilirubin-induced brain injury, language disorder, mood disorders [21], lower IQ [22], and specific learning disorder [22]. Hypernatremic dehydration similarly risks preventable brain damage [23,24], organ failure [25], and death [26]. Neonatal hypoglycemia also risks possible neurodevelopmental harm [27-30], with no consensus definition [31] and an estimated incidence up to 5%-15% of healthy babies [32,33]; like neonatal jaundice, it is also common in less well-resourced countries [34,35]. 

A sizeable minority of breastfed neonates receives inadequate nutrition/hydration [36-39], risking serious injury or death, because breastfeeding insufficiencies including delayed and insufficient milk affect double-digit percentages of mothers. According to some estimates, delayed onset of full milk production (lactogenesis II), defined as mothers perceiving milk coming in beyond 72 hours postpartum, occurs in 33-44% of first-time mothers [39, 40]. In one study of “unusually compliant and well educated” first-time mother volunteers, 15% had persistent insufficient milk after three weeks despite intensive professional lactation support [36]. In that study, minimal prenatal breast enlargement and minimal postpartum breast engorgement as well as prior breast surgery significantly predicted insufficient lactation - suggesting biological root causes. Such insufficiencies put a substantial minority of breastfed babies at risk of suffering insufficient nutrition/hydration for days, weeks, or longer. Such a lengthy deprivation or frank starvation period would normally be considered medically dangerous and legally as well as ethically unacceptable to impose on any human. It overlaps with the riskiest period for neonates, who are most likely to die within their first week of life [41,42]. 

Appropriate supplemental milk prevents possible harm from insufficient milk. What that means varies according to context: Screened and pasteurized milk bank donor breastmilk may be an option in some cases, but supplies are limited and thus access often restricted to preterm and sick infants; other sources of breastmilk (e.g., in wetnursing and shared nursing arrangements where available) pose infectious disease transmission risks. Animal milk may be the most readily available option in some communities, and mothers may prefer it in communities where it has long traditions of supplemental infant feeding use; but it requires fortifying additives and presents special challenges from the act of milking, to transportation, boiling and dilution with boiled water and sugar in the case of cow’s milk, and refrigeration or safe storage in the home [43]. Thus from availability, nutritional, and bacteriological perspectives, formula is usually the best supplemental milk option: It is widely accessible and safe wherever clean water and formula access are sustainable, i.e., absent dire poverty, endemic corruption, and other extreme conditions that can impair basic infrastructure development/maintenance and disrupt essential supplies access. Even under such conditions, these risks may be mitigated, for instance, through context-specific aid and education on boiling or otherwise obtaining appropriate water and other aspects of hygienic preparation; feeding cups, as opposed to plastic bottles with nipples that can be difficult to clean, have long been staples of safe supplemental feeding practices in less well-resourced settings [44], and also have important advantages in several subgroups even in better-resourced settings [45]. Formula further provides more complete nutrition (e.g., including vitamin D) than unfortified animal milks including breastmilk [46]. So while there is no one-size-fits-all solution for every context, harm prevention is as easy as offering hungry infants more milk, and that solution set is widely feasible. Why, then, is insufficient milk intake in breastfed neonates common, frequently missed, and potentially deadly or disabling across country and socioeconomic contexts? 

This article argues that modern misconception of exclusive breastfeeding as natural and thus safe causes common and preventable harm to neonates. Previous advanced civilizations recognized the problem of breastfeeding insufficiencies and built an infrastructure to solve it: Well-organized wetnursing professions [47-51], co- nursing [52], and early infant supplementation (prelacteal feeding) traditions characterized such societies’ infant feeding practices. Widespread prelacteal feeding, seen by some practitioners as helping new mothers rest and newborns stay nourished to promote health and breastfeeding success, continues [53-66], along with more limited wetnursing and co-nursing practices, in many low- and middle-income countries today. 

But modern changes that contributed to increased formula-feeding, such as scientific progress in storing food without spoilage and more women leaving home to work, had killed wetnursing by the early 20th century in Western societies. Intergenerational and other co-nursing arrangements, too, were effectively extinct in these societies by the turn of that century. Then, public health advances dramatically decreased estimated infant mortality in the U.S. between 1850 and 1950: primarily clean water, broader understanding and acceptance of germ theory, pasteurized market milk, and breastfeeding education and encouragement, along with better birth and death registration and continuing technological innovations like pulsating vacuum milking machines that made market milk production cleaner [67-71]. Next, modern Western reformers coming from what had become predominantly formula-feeding societies with sustained intergenerational knowledge gaps about breastfeeding, unaware of the commonality of breastfeeding insufficiencies and of associated serious risks from neonatal starvation, redefined it in terms of exclusivity. This historically anomalous ideal was based on flawed science and logic - and lack of awareness of the negative consequences of such a paradigm shift - and contributes substantially to current infant morbidity and mortality worldwide. 

This review shows that the evidence regarding common and preventable harm to neonates associated with breastfeeding insufficiencies is sufficient to warrant fundamental changes to early infant feeding policies and practices. Current protocols and their implementation, such as the WHO/UNICEF Ten Steps to Successful Breastfeeding codified by the Baby-Friendly Hospital Initiative, emphasize exclusivity in breastfeeding. This emphasis assumes that lengthy neonatal starvation periods are rare when they are common, and safe when they are risky, and that formula (or other best available milk) supplementation necessarily undermines breastfeeding when the evidence is insufficient to establish that claim. Instead, early infant feeding guidance should advise that all neonates be offered adequate milk within two hours of birth and after every nursing session until their mothers’ milk has come in and supply has been established as sufficient, e.g., through typical infant weight gain, behavior, and waste output. 

Default early, adequate, and often formula supplementation is consistent with widespread prelacteal feeding traditions and behaviors worldwide. It applies the precautionary principle to act when grave hazards are possible and stakes are high. At the same time, prior prelacteal feeding traditions were heterogeneous and should be updated according to the best available evidence, including to support safe breastfeeding. This return to prelacteal feeding as the recommended norm would avoid lengthy, risky starvation periods for a substantial minority of neonates. There is insufficient evidence to establish that it would cause harm in turn, including by lowering breastfeeding rates.

## Review

Evidence of substantial, preventable harm to neonates from exclusive breastfeeding

Breastfeeding began its modern resurgence in the mid-1970s, absent the previous safety infrastructure including a well-organized wetnursing profession and prelacteal feeding traditions that had protected many neonates from harm from common breastfeeding insufficiencies; and evidence of associated harm began accumulating shortly thereafter. Beginning in the late 70s, Gilmore and Rowland [72], Roddey et al [73], Rowland et al [74], and Thullen [75] reported cases of severe malnutrition in breastfed infants, usually associated with primapara mothers and neonatal jaundice, including significant clinical sequelae such as profound cardiovascular collapse, severe hypernatremia, perforated duodenal ulcer, and transient renal failure, all of which may develop in response to acute starvation. Gilmore and Rowland noted all affected infants were first children of upper middle class parents, while Rowland et al emphasized that malnutrition from breastfeeding insufficiencies may progress rapidly and not be recognized by “otherwise intelligent,” motivated parents - sentiments echoed by clinicians today. 

Breastfeeding’s resurgence, still without infrastructural protections against harm from common insufficiencies, continued. Since before “The Baby Killer” report [76] triggered the Nestlé Boycott and 1978 Senate hearing, doctors (including those the report cited) tended to dismiss mothers’ double-digit self-reports of insufficient milk. Researchers (with good intent) created datasets that made it impossible to empirically assess such claims, reproducing widespread dismissal of women’s perceptions of common breastfeeding insufficiencies. Neonates suffered the consequences. As Neifert and Seacat observed [77], ”The overenthusiastic promotion of unsupplemented breastfeeding” even in cases with clear risk factors for breastfeeding insufficiencies ”can lead to critical failure to thrive in the infant.” 

By the mid-90s, a rise in associated preventable newborn hospitalizations was noted, along with recurrence of kernicterus [78-80], which disproportionately affects Black babies [81]. Edmonson et al [82], Paul et al [83], Flaherman et al [84], and others found exclusive breastfeeding correlated with preventable newborn readmissions for starvation effects including dehydration and jaundice. Kemper and McCarthy [85] found breastfeeding was substantially more common among infants who developed jaundice. Koziol et al [20] noted breastfeeding was a risk factor for kernicterus, and posited subcortical mechanisms of the related spectrum of bilirubin-induced neurologic dysfunction (BIND or partial kernicterus syndromes) in infants who developed even moderate elevations in bilirubin. Amin et al [22] reviewed the observational evidence linking the BIND spectrum and comorbid neurodevelopmental disorders including cognitive delay, attention deficit hyperactivity disorder, specific learning disorder, autism, and/or language disorder, delineating multiple possible biological mechanisms for the link. 

Still, breastfeeding proponents dominant in most medical and public health institutions concerned with early infant feeding continued to strive to increase exclusive breastfeeding - without infrastructural safeguards, relevant safety data, or informed consent regarding possible risks. So, too, evidence of associated harm continued increasing. Beginning in the late 90s, Cooper et al [86], Laing and Wong [87], Moritz et al [88], and Reilev et al [89] showed breastfeeding-associated hypernatremia (often comorbid with jaundice) was increasing. Moritz and Ayus [90] warned of its non-specific symptoms and potentially lethal or disabling progression, echoing Koh et al’s [28] warning that neonates with neural dysfunction during hypoglycemia often appear asymptomatic. Reilev et al showed risk of hypernatremic dehydration increased only with degree of relative weight loss, warning the rise could be associated with Baby Friendly, the WHO/UNICEF program of exclusive breastfeeding promotion which educates mothers on benefits but not risks of exclusive breastfeeding and discourages supplementation unless “medically necessary” - a condition lacking consensus definition. The WHO/UNICEF continued, then as now [91], to discourage formula supplementation and make no mention of exclusive breastfeeding’s risks as part of its Ten Steps to Successful Breastfeeding, Baby Friendly, and related breastfeeding promotion programs. Neifert warned again [92] that ”Those who enthusiastically promote breastfeeding... must confront the reality of breastfeeding failure and implement necessary changes in medical education and support services to foster successful outcomes in breastfed infants” who were otherwise in danger of failure to thrive and hypernatremic dehydration from insufficient milk intake. 

Such warnings went unheeded, and evidence of associated harm continued mounting. Moritz et al [88] found 1.9% of healthy term and near-term breastfed babies at Children’s Hospital of Pittsburgh were admitted for breastfeeding-associated hypernatremic dehydration, with the vast majority (87%) first-borns and most (81%) presenting with jaundice. Flaherman et al [84] found exclusive breastfeeding increased newborn hospitalizations by 2.2% and also correlated with more outpatient visits before hospitalizations: Concerned caretakers sought medical help, but neonates still went hungry. Mette et al found healthcare providers admitted less than half their sample, almost none of whom were supplemented, suggesting widespread problems in medical recognition. Futatani et al [93] found 37% of exclusively breastfed, full-term singletons at Toyama Prefectural Central Hospital lost at least 10% of their birthweight, of whom 20% had blood ketone levels beyond the diabetic ketoacidosis threshold. Ferrández-González et al [94] found a 30.9% incidence of hypernatremia in near-term and term neonates at the Vega Baja Hospital (Orihuela, Alicante, Spain), with 74.5% of hypernatremic infants exclusively breastfed. Shan et al [95] found rooming-in was associated with increased exclusive breastfeeding, birthweight loss >10% at day 3 of age, and neonatal admission for phototherapy for hyperbilirubinemia; mixed-fed neonates also saw hospitalization risk increase, suggesting even supplemented neonates may frequently suffer medically inadequate nutrition/hydration under Baby Friendly. Discouraging formula supplementation to promote exclusive breastfeeding comes at the cost of risking possible harm to neonates.

Time matters

These findings suggest a neonatal starvation problem of considerable scale, underestimated by hospital postpartum readmission data that exclude diagnoses like failure to thrive, growth faltering, wasting, and underweight that often avoid hospitalization, diagnoses during continued birth hospitalization, and missed diagnoses. Jaundice is likely underdiagnosed for many reasons, including substantial possible false-negative bias in the standard neonatal jaundice diagnostic tool [96-98]. The true scale of the problem is unknown and uncertainty pervades existing knowledge about associated risks; but time matters. 

Insufficient nutrition worsens jaundice as bilirubin builds up with significantly decreased voiding in breast- versus formula-fed infants [99]. Progressive dehydration worsens hypernatremia. Hypoglycemia worsens as cellular stores of glucose deplete. There is no established safe level of neonatal hyperbilirubinemia, hypernatremia, or hypoglycemia. Even moderate hyperbilirubinemia [20] and moderate, early, and transient neonatal hypoglycemia risk neurodevelopmental impairment [29]. 

Counter-intuitive patterns of apparently disparate risk between preterm and near- or full-term infants are consistent with the possibility that an alternative early infant feeding paradigm limiting neonatal starvation periods could prevent a large number of deaths and disabilities. On one hand, a vast body of literature has long established that preterms are more vulnerable to many adverse outcomes including jaundice. On the other hand, preterms appear less vulnerable to possible neurodevelopmental harm from consequences of early insufficient milk intake. Neonatal hypoglycemia increases term infants’ autism risk threefold but does not increase the risk in preterms [100]. The same pattern of possible lower or no increased risk for preterms as opposed to full-term neonates also holds for jaundice and autism risk [9-11]. Why would a more vulnerable subgroup show less vulnerability to neurodevelopmental harm? 

This counter-intuitive pattern is consistent with the possible explanation that the problem is insufficient milk intake, and time matters. Preterms’ heightened medical monitoring may result in earlier detection of complications from insufficient milk intake. Their heightened vulnerability may result in earlier intervention. Both may help prevent neurodevelopmental harm from insufficient milk intake. Different norms surrounding supplementing preterms early and often with adequate milk may also contribute: Preterms often have greater access to limited donor breastmilk, and are typically fed with fortified milk regardless of milk type, increasing their caloric and vitamin intake. Mothers of preterms are widely recognized as having a higher frequency of insufficient milk, flagging preterms for likely supplementation need. Another possible contributing factor is survivor bias: Preterms are at heightened risk of death from many causes and might also die from causes relating to neonatal starvation at higher rates than full-term neonates, leading them to suffer comparatively less associated neurodevelopmental harm later. 

In the time it often takes mothers’ milk to come in, their neonates risk death or permanent disability. Because time seems to be one important factor in the potential severity of complications from insufficient milk intake, minimizing neonatal deprivation or starvation periods by providing sufficient milk may prevent substantial harm to neonates. But current guidelines fail to recognize the commonality and severity of these possible harms, as well as the importance of time in mitigating associated risks.

Conflict between current guidelines and evidence

Current guidelines recommend waiting to offer formula at least until the medical need has been established, treating formula-feeding as riskier than starvation or invasive medical intervention. The Academy of Breastfeeding Medicine (ABM) 2017 supplementation guidelines indicate supplementation when birthweight loss exceeds 8%-10% at day 5 or later, or the 75th percentile for age, and then only after “a thorough evaluation” [101]. ABM’s 2021 hypoglycemia guidelines [102] ignore starvation as a possible cause of neonatal hypoglycemia, although it is likely the most common cause. They also de-emphasize formula-feeding as the last possible supplementary feeding option among many, listing “extra expressed breastmilk, pasteurized donor human milk, or infant formula (with your permission),” as if formula carried special risks that parents might want to avoid, although such risks are not established and no other option is usually available for healthy, near- or full-term neonates when breastfeeding is insufficient. Similarly, Pediatric Endocrine Society recommendations for high-risk neonates “suggest evaluation when the infant is ≥48 hours of age so that the period of transitional glucose regulation has passed [103]” - contradicting evidence that moderate neonatal hypoglycemia starting within six hours of birth elevates risk of developmental delay at 2-6 years [30]. 

Baby Friendly recommends breastfeeding initiation as soon as possible after birth, which is compatible with nursing followed by formula supplementation until breastfeeding is established. Hofvander [104] found that among Swedish Baby Friendly hospitals, some routinely supplemented less than 10% of healthy neonates until breastmilk came in, while in others up to 90% were supplemented. There seems to be little available evidence on such hospital-level variation in interpreting and applying Baby Friendly-style exclusive breastfeeding promotion. But existing evidence is insufficient to support the idea of a forced choice between supporting breastfeeding - including by denying hungry neonates formula - and preventing harm to neonates from insufficient nutrition/hydration by offering early, adequate, and often formula supplementation instead. 

In a set of randomized trials, researchers reported conflicting effects of formula supplementation on breastfeeding. Flaherman et al [105] (N = 40) found early limited formula supplementation decreased formula use at one week and three months. But the apparent increase in exclusive breastfeeding from such supplementation failed to replicate; one possible explanation for the initial result is chance, likely relating to small sample size. In the later, larger trial (N = 164), Flaherman et al [106] reported the reverse effect: Investigators observed decreased exclusive breastfeeding among infants who had received early limited formula, and the effect reached statistical significance at infant age 12 months and when any breastfeeding (as opposed to exclusive breastfeeding) was considered [107]; breastfeeding duration at 12 months was the planned primary outcome measure according to the clinical trial register (ClinicalTrials.gov). 

The authors noted post hoc that the intervention group had lower intended breastfeeding duration on study entry than the control group, potentially explaining the difference. But the study design had blocked on location and method of delivery to account for potentially relevant variation in the randomization procedure, at which time researchers did not consider maternal intent relevant. Inconsistent findings such as these leave open empirical questions about the effects of formula supplementation on breastfeeding, as well as highlighting the potential methodological danger of researcher degrees of freedom influencing results [108]. These trials also raise troubling ethical questions about apparent lack of adequate protection for vulnerable subjects, as the exclusively breastfed neonates studied were identified as having already lost substantial weight, but randomized to receive either very limited or no supplementation; preventable hospitalizations resulted. 

Harm prevention is as easy as giving a bottle. Formula lowers bilirubin by inhibiting intestinal reabsorption of it [109], treats hypoglycemia with appropriate protein, fat and carbohydrate supplementation, treats dehydration with additional fluids, and ensures nutrient status. Supplementation with water or sugar water does not reduce hyperbilirubinemia [110,111], treat dehydration with appropriately balanced fluid, or provide nutrients, and may create blood sugar spikes that worsen hypoglycemia later. Thus Rozance et al [31] note ”Milk feedings particularly enhance glucose homeostasis.” By contrast, treatment options such as phototherapy and exchange transfusions for jaundice, or IV glucose or glucose gel with hospital monitoring for hypoglycemia, are invasive, costly, potentially stressful for neonates and their families, and may incur substantial risks. In infants who are already medically endangered by insufficient milk intake, these interventions may be necessary to limit brain injury and disability, for example by swiftly correcting critically low glucose with oral dextrose in addition to supplementation; again, time matters. But these cases are overwhelmingly preventable through supplementing breastfeeding with formula in response to signs of persistent infant hunger at the latest. Formula-feeding in places with clean water, high literacy, and stable formula access is well-established as safe. Generations of healthy humans have already been formula-fed from birth, and formulations of formula have improved since that was the norm with substantial nutritional advances (e.g., the additions of choline and omega-3 fatty acids). 

The comparative safety advantage of prelacteal feeding over exclusive breastfeeding may be greater in relatively resource-poor settings. In lower-income countries, starvation effects are likely to also include delayed diagnosis and treatment, resulting in excessive use of belated (ineffective and risky) exchange transfusions [112] for severe hyperbilirubinemia in the same countries disproportionately affected by associated permanent disability and death [113]. As Slusher et al’s review [113] detailed, harm associated with severe neonatal jaundice in low- and middle-income countries presents context-specific measurement problems (e.g., limited data). But existing evidence is sufficient to establish that ”Acute bilirubin encephalopathy (ABE), exchange transfusions and death are frequent and costly outcomes of severe neonatal jaundice (SNJ) especially in low-income and middle-income countries.” Similarly, Cayabyab and Ramanathan’s review [114] noted that ”Severe hyperbilirubinemia occurs more frequently in infants from low- and middle-income countries (LMIC),” while exclusive breastfeeding should be but is often not recognized as a risk factor. This suggests that, even where formula-feeding may be associated with heightened risks (e.g., due to unclean water and formula access disruption), those risks may be outweighed for affected infants by the risks of neonatal starvation associated with breastfeeding insufficiencies in the absence of safety infrastructure. If public health authorities and NGOs promoting exclusive breastfeeding’s benefits without mentioning its risks do so in lower-resourced settings on the basis of explicit, evidence-based calculations showing that, under a different policy regime, more neonates would likely die or be disabled from unhygienically prepared or diluted formula supplementation than die or are disabled from insufficient milk intake associated with breastfeeding, then these calculations have been private; relevant data and analyses should be subjected to public scrutiny, and the implicit premise that some infants must starve in order to save others should be subjected to democratic oversight. 

Additional evidence suggests that exclusive breastfeeding promotion in the absence of safety infrastructure addressing common breastfeeding insufficiencies may be associated with increased infant morbidity and mortality in less well-resourced settings. For instance, the PROMISE-EBF trial (Promoting Infant Health and Nutrition in Sub-Saharan Africa: Safety and Efficacy of Exclusive Breastfeeding Promotion in the Era of HIV), conducted from 2006-2011 in Burkina Faso, Uganda, South Africa, and Zambia reported some findings suggestive of possible increased neonatal mortality risks associated with breastfeeding insufficiencies in the context of heightened emphasis on exclusivity in the intervention arm of the trial: Diallo et al [115] reported the highest-ever measured perinatal mortality rate in Burkina Faso. Diallo et al [116] reported maternal nulliparity significantly predicted neonatal death (95% CI 1.5 to 12.1), consistent with a possible link with breastfeeding insufficiencies. 

One alternative, not mutually exclusive explanation is that lack of medical care contributed to prolonged labor generating birth trauma resulting in stillbirths and neonatal deaths on days 0-2 of life (T. Tylleskär, personal communication, September 16, 2021). But it is not clear why obstetric neglect would have increased during the trial. Trial data cannot be used to test either hypothesis, because information on causes of death, possible complications resulting from insufficient milk intake, or maternal perceptions of breastfeeding problems were not collected. 

Engebretsen et al [117] reported the PROMISE study intervention successfully decreased prelacteal feeding in Burkina Faso. Engebretsen et al [118] reported this intervention was associated with more wasting in Uganda and lower ponderal growth in Uganda and Burkina Faso. Investigators hypothesized these apparent adverse effects may have resulted from misguided public health and medical (including PROMISE) encouragement to mothers to continue exclusively breastfeeding for six months, when infants may need supplementary foods beginning around four to six months to ensure appropriate nutrient and caloric intake. This is consistent with Forsyth’s criticisms [119] that prolonged exclusive breastfeeding leads to later child mortality and morbidity, a six-month recommendation is too rigid, and context-specific interdependence of breastmilk, complementary foods, and infant formulas if required should be central to early infant feeding research and policymaking. It is worth noting that the PROMISE team’s possibilities were constrained in this context by the current exclusive breastfeeding promotion regime, which since the World Health Assembly’s 1981 adoption of the routinely amended International Code of Marketing of Breast-milk Substitutes (the WHO Code) [120] has increasingly discouraged donations of free or low-cost formula and other supplies to healthcare facilities, even in settings with endemic poverty and communicable diseases like HIV that are transmissible through breastfeeding. Thus formula is unavailable in many low-resource settings even when it is medically necessary. 

Reported PROMISE results showed no possible infant health benefits to balance the potential risks of lengthening neonatal starvation and later infant under-nutrition periods through the study’s exclusive breastfeeding promotion. Tylleskar et al [121] reported the intervention did not significantly affect the prevalence of diarrheal disease. Tumwine et al [122] reported a possible decrease in general cognition scores (95% CI -0.40 to 0.05). Investigators interpreted these findings as null, and hypothesized that they might be explained by the high prevalence of exclusive breastfeeding across treatment and control groups. They might also be partly explained by different subgroup effects; for instance, some infants might have become more vulnerable to diarrheal disease when they received insufficient nutrition/hydration due to breastfeeding insufficiencies, while others might have become less vulnerable when breastfeeding worked early and well. 

Unaffiliated with PROMISE, Bhattacharjee et al reported the estimated national exclusive breastfeeding prevalence in Burkina Faso nearly doubled between 2000 and 2017 [123]; this increase coincided with the country’s highest-ever measured perinatal mortality rate during PROMISE. Overall, the evidence suggests that exclusive breastfeeding promotion, in the absence of safety infrastructure protecting infants from common breastfeeding insufficiencies, may have contributed to preventable infant mortality associated with lengthier neonatal starvation periods in sub-Saharan Africa, as well as to worse developmental outcomes including growth and cognition. But researchers neither collected data on breastfeeding insufficiencies that would enable relevant analysis, nor mentioned related risks in the study protocol, instead describing breastfeeding unequivocally as safe - a typical representation in current research and policy. That representation ignores evidence that a sizeable minority of mothers will experience breastfeeding insufficiencies likely to cause serious clinical problems in some of their exclusively breastfed infants; in less well-resourced settings such as these, those infants are relatively highly likely to die or suffer permanent disability as a result. Why, then, would trials such as this still be widely considered ethical? 

Current thinking about early infant feeding assumes exclusive breastfeeding benefits infant health without incurring meaningful risks. But that paradigm is based on flawed science and logic. The science is vulnerable to Greenland’s criticism that standard categorical analysis neglects to account for within-category information [124]. Widely recognized potential confounds for exclusive breastfeeding’s purported infant health benefits include breastfeeding insufficiencies as well as maternal health and socio-economic status [125-127]. 

For example, Clavano’s compelling 1978 U.S. Senate ”Nestlé” hearing testimony [128] on the benefits of breastfeeding according to her research in the Philippines avoided mentioning that, while the shift from formula to breastfeeding in her hospital correlated with dramatic decreases in neonatal morbidity and mortality, it also correlated with a dramatically shortened “starvation period [129].” That period was 8-12 hours for normal babies and 16-24 hours for preterms, gradually shortened to 8, then 6, then 4, and finally 2 hours, for a 6-22 hour shortening of neonatal starvation periods. This does not establish breastfeeding benefits or formula risks. Rather, it might be interpreted as evidence that feeding neonates within the first few hours of birth, instead of waiting up to almost a full day in some cases, benefits their health: Neonatal starvation may carry considerable risks. The research providing sufficient detail to allow the reader to draw that alternate conclusion does not appear in the Congressional record. 

Similarly, the largest breastfeeding trial ever conducted, Promotion of Breastfeeding Intervention Trial (PROBIT) [130] - a cluster randomized experiment assigning freshly post-Soviet Belarusian hospitals to Baby Friendly-style treatment or control groups - neglected to collect data on relevant post-randomisation events. This is a problem because women who supplemented their infants with formula sooner rather than later may have done so for clinically relevant reasons including delayed and/or insufficient milk. Delayed onset of lactogenesis II predicts excessive neonatal weight loss, formula supplementation, and earlier breastfeeding cessation [36,40], while insufficient milk has long been among the most commonly cited reasons for early weaning worldwide [131-135]. Thus breastfeeding problems likely systematically biased the formula-fed group in this and other studies to contain more babies who had endured longer starvation or deprivation periods, which may have influenced all later outcomes of interest. Subgroup variation in inadequate nutrition/hydration might explain apparent differences between exclusively breastfed and mixed- or formula-fed infants, including reported differences in growth, infections, allergic diseases, and IQ. 

For example, overlapping inverse effect estimates for exclusive breastfeeding and failure to thrive in relation to IQ are consistent with this possibility. Hospital-level randomization to Baby Friendly in PROBIT increased exclusive breastfeeding at 3 months (43.4% versus 6.4%) and subsequently correlated with child Wechsler IQ scores at age 6.5 of an average -1 point decrease to 12.8 point increase [136]. These confidence intervals overlap with inverse estimated changes in Wechsler scale IQ scores at 8 years associated with failure to thrive in the first 2 months according to the Avon Longitudinal Study of Parents and Children [137]. The latter highly statistically significant, linear effect averaged around three points. Both PROBIT and Avon sets of estimated cognitive effects were strongest for verbal IQ. 

While IQ is not diagnostic of autism, verbal and other communication problems are typical of it; and three meta-analyses found neonatal jaundice may substantially increase autism risk [9-11]. More broadly, Amin et al [22] review the evidence on unconjugated hyperbilirubinemia and neurobehavioral disorders, and report ”Although the association between UHB [unbound hyperbilirubinemia] and cognitive delay is biologically plausible, data regarding the association between UHB and cognitive delay, that is, lower measured intelligence quotient (IQ) relative to same-age peers, are conflicting.” Lower IQ is a possible neurodevelopmental harm associated with neonatal jaundice, which in previously healthy, near- and full-term breastfed neonates is usually associated with insufficient milk intake. 

IQ effect estimates from PROBIT and Avon also overlap estimates of the so-called Flynn and reverse Flynn effects of societal-level IQ level increases and then decreases [138-140]. Bratsberg et al [141] suggested the Flynn effect was environmentally caused; Lynn et al [142] agreed, proposing that Flynn effect IQ gains probably resulted from improvements in pre-natal and early post-natal nutrition. Future research should examine the relationship between Flynn and reverse Flynn effects and early infant feeding norms, particularly with attention to the possibility that subgroup effects matter. Exclusive breastfeeding promotion may have first caused aggregate societal IQ gains where it reduced average neonatal starvation periods, but then caused aggregate losses as continuing increases in exclusive breastfeeding grew the subgroup of the most adversely impacted neonates - those for whom breastfeeding did not work early or well, but whose mothers complied with ever-increasing pressures to keep doing it anyway. This would result in a parabolic relationship between societal-level IQ changes and exclusive breastfeeding rates. In other words, one might expect two differently valenced effects to appear in sequence as aggregate gains from shorter neonatal starvation periods were replaced over time by aggregate losses from greater exclusive breastfeeding pressure on mothers for whom breastfeeding does not work fast and /or well. The latter increasing pressure would likely work in combination with weaker societal formula-feeding norms over time. Thus changing early infant feeding norms may have produced at least part of the observed pattern of both Flynn and reverse Flynn effects. This appears to be the only proposed explanation for a single causal contributor to both parts of this pattern. 

Baby Friendly achieved important reforms including likely shortening average neonatal starvation periods by promoting rooming-in and early breastfeeding initiation. These new (contemporary) norms replaced old (modern) norms including neonates being routinely taken from new mothers without their consent, and formula-fed - sometimes after lengthy starvation periods - in full hospital nurseries in which they were additionally more vulnerable to the spread of disease and lacking in the one-on-one care that is more likely in a familial context. But in terms of established medical benefits and risks to neonates in particular settings, the effects of Baby Friendly remain largely uncertain. Data on prior starvation periods and related complications do not seem to be widely available; bloc (Soviet versus Western), geographic, country, cultural, hospital, hospital staff, and familial practices were likely heterogeneous. Future research might examine relevant data from former Soviet bloc hospitals in a difference-in-differences analysis, since supplementation was a strong Soviet norm across countries, and base rates of possible complications from insufficient milk intake such as hyperbilirubinemia may have been recorded before and after the shift to Baby Friendly. However, data problems from the chaos of the collapse of formerly Soviet societal structures that went hand in hand with adoption of Western practices like Baby Friendly may preclude this type of analysis. 

Bracketing that sort of empirical question, even if we accept that the Baby Friendly shift produced aggregate gains, the evidence shows that it simultaneously lengthened starvation and deprivation periods for some neonates. Lack of safeguards against breastfeeding insufficiencies harmed infants who disproportionately went on to be formula-fed as caretakers acted (against earlier medical advice) on cues that these infants were going hungry. This produced what may look like evidence that breastfeeding benefits children’s neurodevelopment, but actually be additional evidence that insufficient nutrition/hydration during the sensitive early postnatal period harms it. 

Such flawed science combined with the naturalistic fallacy to promote exclusivity in breastfeeding even when it meant babies went hungry, as Baby Friendly promoted new practices without safety infrastructure or data collection relating to common breastfeeding insufficiencies. Recent research suggests serious additional - albeit much less common - associated risks beyond insufficient milk intake, including from in-hospital falls relating to maternal fatigue due to round-the-clock early breastfeeding efforts [143], neonatal collapses relating to prone positioned early breastfeeding in primaparas [144], and co-sleeping harms [145]: What was presumed natural, and thus safe, is neither. 

These risks highlight additional possible benefits of old breastfeeding practices over new: Letting mothers rest for the first few hours or days after birth might avoid the documented increase in falls by decreasing the maternal fatigue associated with round-the-clock breastfeeding efforts that appear to be their root cause. Waiting to initiate breastfeeding for the first few hours or days postpartum while supplementing early and often with formula might avoid the documented increase in potentially lethal or permanently disabling neonatal collapse events by protecting more physiologically vulnerable neonates from weakening due to insufficient or absent nutrition/hydration combined with positional suffocation and other physiological stressors. In addition to mitigating more direct and common possible harms from insufficient milk intake reviewed above, feeding hungry neonates formula would also likely reduce these relatively rare but serious events. 

Executing the indicated paradigm shift to feeding hungry neonates supplemental milk as a risk-mitigating default would generate a new research agenda. Early, frequent suckling may promote milk production [146]; but sending that signal through expressing, pumping, and suckling may be compatible with or even enhanced by letting mothers rest and neonates drink formula as needed. Future research should compare related interventions to learn more about how to best support safe breastfeeding. Reinstating norms where mothers have access to help with nursery care to rest immediately postpartum while neonates are supplemented early and often with adequate formula is likely to also support separate sleeping arrangements from the beginning; this might decrease risky co-sleeping practices associated with nursing on demand (round-the-clock breastfeeding in response to neonates’ hunger cues). Future research should investigate how to best support such safer sleeping practices. Above all, revised early infant feeding guidelines must emphasize informed consent, recognizing the risks and benefits of all options rather than prescribing a one-size-fits-all approach. At the same time, societal decisions about harm prevention always place limits on individual freedom; and the evidence suggests that widespread problems of lack of recognition among parents and medical professionals alike may contribute to common and preventable harm to neonates associated with breastfeeding insufficiencies. Future research should identify best practices in empowering all caregivers to make informed early infant feeding decisions and recommendations that optimize each individual infant’s health outcomes, never prioritizing a public health statistic (i.e., exclusive breastfeeding rates) over an individualized clinical judgment - or a misconception over an infant’s safety. 

Experts must reconsider formula-feeding as an infrastructural innovation to help keep newborns safe, on par with clean water. Neither intervention is natural; both are logical. Reinstating the other infrastructural solutions history offers - a well-regulated wetnursing profession and co-nursing practices - are riskier now due to transmissibility of infectious diseases such as HIV, but could also be explored as possible public health interventions to increase neonates’ early breastmilk intake while decreasing risks from breastfeeding insufficiencies. They might have the added advantage of making safe infant feeding more resilient in the face of potential supply chain disruptions from increasingly likely extreme weather events, pandemics, and other forms of instability related to climate change. But recreating such an infrastructure would also take considerably more resources than recommending early, adequate, and often formula supplementation, a simple solution that applies available evidence to minimize fully preventable harm to neonates now. 

Current guidelines including from the American Academy of Pediatrics and International Lactation Consultant Association recommend maximum neonatal weight loss of 7%, a figure based on mean weight loss without accounting for standard deviation [147]. This threshold includes values that risk death and disability in previously healthy, full-term neonates. Hypernatremia has been reported with neonatal weight loss <4.8% [94], and profound hypoglycemia with only 4.5% [148]. In recent experiments, too little or no supplementation in neonates with weight loss ≥5% but <10% of their birthweight at 24-48 hours old [105] or who were in the ≥75th percentile for weight loss at age at 24-72 hours [106] was associated with preventable hospitalizations for hyperbilirubinemia. Chantry, a past President of the Academy of Breastfeeding Medicine, had publicly criticized the appropriateness of this design before Flaherman et al attempted to replicate their earlier results [149]; documents obtained under the Freedom of Information Act show that investigators neither informed their Institutional Review Board of these risks, nor reported the adverse events to the U.S. Department of Health & Human Services, which funded the second trial [150]. 

Zhao et al [151] recently found breastfed newborns who lost >4.5% of their birthweight in the first 24 hours had significantly lower serum bilirubin when supplemented, and early and often formula supplementation worked best. Hensman et al [152] similarly found delivery by C-section (a likely proxy for formula-feeding) and every single milliliter of formula given for the first three days was protective against readmission of healthy term neonates. Hunt et al [153] reported reducing a medical center’s neonatal jaundice readmission rate from 2.6% to .8% through a jaundice management guide that included applying new infant supplementation guidelines by adding standardized newborn weight collection at 24 hours of life, a change they state ”improved neonatal care, even for those without jaundice,” suggesting that weighing all neonates at 24 hours of life identified more neonates in need of supplementation than could have been identified through jaundice diagnosis alone. Conversely, Grupp-Phelan et al [154] replicated others’ finding [1,82,83] that lengthier hospital stays do not matter much, calculating that “One hundred twenty-two infants would have to stay for longer than 30 hours to avoid 1 jaundice readmission.” Formula supplementation alone, not more medical care or better monitoring, could prevent most U.S. neonatal readmissions. These findings echo Okawa et al [155], who in 1988 noted that breastfed infants who received insufficient milk had lower fluid and caloric intake, greater weight loss, and potentially associated higher incidence of hyperbilirubinemia and need for phototherapy, while formula supplementation appeared to improve all these outcomes. 

More or better measurement is not the answer. Medical monitoring using weight thresholds will always risk preventable harm, because subgroup calculations are complex, and the datasets used to build technology to help calculate these risks are biased toward WEIRD [156] (Western, Educated, Industrialized, Rich, and Democratic) societies. This limitation applies equally to stationary percent thresholds based on mean weight loss, which fail to account for dispersion, and to comparisons of a neonate’s weight loss to that of a reference cohort, estimating percentiles of weight loss as a function of time from birth, like the free website Newt (newbornweight.org) does using a cohort of around 160,000 California neonates [157]. For jaundice, a lower threshold of concern is appropriate in the context of: prematurity [158]; low birthweight; systemic infection such as sepsis; birth trauma [113]; C-section; male gender [159]; Asian race due to increased incidence of UGT1A1 [160-162]; African, Sephardic Jewish, Greek, Turkish, Chinese, and Italian race due to increased risk of glucose-6-phosphate dehydrogenase (G-6-PD) deficiency [163]; and darker skin due to melanin reducing visible skin yellowing [164]. Technology assisting these subgroup risk assessments cannot be built using currently existing data and would have to grapple with complex questions including how to code mixed-race or intersex neonates. The simpler and better, default harm-preventing protocol is feeding hungry neonates formula. 

Other proposed technology to assist these assessments includes simple devices that measure bilirubin (TSB) [114], which has high sensitivity but low specificity for associated brain injury [22]. Measuring unbound unconjugated bilirubin (UB) is more predictive but more technically difficult, [165] and so unlikely to become part of routine care, particularly in less well-resourced settings that appear to disproportionately bear the burden of increased infant morbidity and mortality from breastfeeding insufficiencies. Moreover, there is still no established safe level of elevated UB vis-a-vis neurodevelopmental harm. The problem of differential and unknown subgroup vulnerabilities persists here, too. Again, formula supplementation presents a simpler, safer alternative. 

Available evidence establishes currently estimated thresholds for excessive early infant weight loss as unsafe and poorly conceived, and estimating safer thresholds as theoretically unjustified. Neonatal starvation and later infant under-nutrition have no established benefits, grave possible risks, and easy prevention. Lower thresholds for what constitutes excessive early infant weight loss would still lead to fully preventable harm in previously healthy, near- and full-term neonates. Evidence on differential subgroup vulnerability suggests that this harm would disproportionately affect male neonates, those of Asian and African descent, and those with poorer medical care access - sometimes resulting in death or lifetime disability. These are unacceptable disparities in unacceptable outcomes. A different early infant feeding paradigm is urgently needed.

Discussion

In no other advanced civilization have neonates across development and socioeconomic strata routinely risked death and disability as a result of inadequate nutrition/hydration while healthcare professionals actively discouraged supplemental feeding. Rather, before ours, such civilizations had an infrastructure (i.e., wetnursing, shared nursing, and prelacteal feeding) to prevent harm associated with common breastfeeding insufficiencies. Modern societies accidentally destroyed that infrastructure, which modern conditions (e.g., infectious disease risks and restrictions on selling bodily materials or functions) pose challenges to recreating. Then modern societies reintroduced breastfeeding in terms of a new, historically anomalous practice emphasizing exclusivity but lacking in safety infrastructure protecting against breastfeeding insufficiencies, while de-prioritizing sufficient feeding and prevention of infant hunger, dehydration, and even acute starvation. 

Before that reintroduction, modern prevention of harm from neonatal starvation entailed switching breastfed babies who developed jaundice to the bottle. That was the clinical practice norm that exclusive breastfeeding promoters replaced without safety data on their new intervention, which they misconceived as natural and thus safe. The absence of any system of public reporting on rates of insufficient feeding complications keeps patients from making informed choices regarding the hospital policies with which they wish to participate. Early, adequate, and often formula supplementation of breastfeeding during the high-risk period immediately postpartum remains the most viable and well-established solution to the perennial and common breastfeeding insufficiencies problem. 

While evidence-based, this view departs from current consensus spanning professional medical organizations, national-level public health authorities, and non-governmental organizations as well as myriad civil society interest groups such as lactation consultants and breastfeeding activists. That disconnect situates exclusive breastfeeding promotion in the realm of mainstream medical procedures and public policies without sufficient scientific basis and thus safety data - many of which have been proven to not work or even to backfire. For example, in medicine, formerly common arthroscopic surgery for degenerative knee disease appears to confer no long-term benefits in pain or function [166,167]. In public policy, formerly widespread U.S. teen substance abuse prevention program D.A.R.E. (Drug Abuse Resistance Education) did not work to systematically reduce teen drug use and may have even increased it [168-170]. At best, such cases reflect greater need for evidence-based, public interest oversight in medicine and health policy. At worst, they illustrate the larger crisis in science, beyond statistical methodological concerns like p-hacking (manipulation of significance testing to find positive results) [108] and reproducibility [171], in which rampant fraud and mistakes encouraged and then hidden within the “publish or perish” incentive system undermine the knowledge system that is supposed to underpin medicine and public policy [172-176]. What would each of these possibilities mean for mitigating common and preventable harm to neonates from breastfeeding insufficiencies? 

Regarding oversight, there is a double standard in risk mitigation when it comes to breastfeeding. Professional associations, state public health actors, and NGOs treat other serious risks to infants very differently from how they treat risks of common and preventable harm to neonates from breastfeeding insufficiencies. For example, infant botulism and Sudden Infant Death Syndrome (SIDS) are both extremely rare [177, 178], but have been the subjects of extensive preventive efforts by groups like the American Academy of Pediatrics, the WHO, the U.S. Centers for Disease Control and Prevention, and analogous organizations. Parallel efforts to inform expectant and new mothers about the far more common risks of breastfeeding insufficiencies in order to prevent associated harms have not been forthcoming from the same organizations, which instead uncritically promote exclusive breastfeeding today as they have done for decades despite mounting evidence of serious risks. Even formula labels state - incorrectly in the case of breastfeeding insufficiencies - that breastfeeding is best for babies. Against this consensus chorus, efforts to address preventable harm to neonates from breastfeeding insufficiencies as a public interest oversight problem in medicine and health policy have been spearheaded by volunteer organizations like the U.S. Fed Is Best Foundation [179] and U.K. Infant Feeding Alliance [180] (inspired by Fed Is Best) - both of which are led by mothers whose babies suffered accidental starvation at their breasts. 

Any of these organizations, from professional associations to NGOs, might lobby their respective country’s institutions to review the evidence on early infant feeding. For example, the American Academy of Pediatrics could ask the U.S. Congress to call on the Government Accountability Office (GAO) and National Academy of Medicine (NAM) in this task. GAO reviews relevant federal government activities when there are allegations of waste, fraud, and abuse, and the National Academies review scientific evidence. Both have a history of assembling investigators and committees without conflicts of interest to produce reports that contribute to necessary policy regime changes. By focusing on the national level, these reviews could be both comprehensive and context-sensitive. They might lead to changes in guidelines which conflict with available evidence. No organization has yet publicly called on such institutions for these services. Thus, targeted grassroots lobbying efforts may be one answer to the policy problem of insufficient evidentiary oversight of early infant feeding policies and practices. 

Addressing the crisis in science starts with the same step. A comprehensive review of the existing evidence would audit study quality, clarifying where apparent mistakes and/or possible fraud may have compromised the integrity of the publication record, estimating risks and benefits of different early infant feeding paradigms, and highlighting likely biases and gaps in the literature. It would note that, to date, no large-scale breastfeeding trials have been conducted that include data on relevant post-randomization events including breastfeeding problems. It would suggest a future research agenda. 

The missing gold standard evidence on breastfeeding’s benefits and risks is experimental. Breastfeeding proponents argue it would be unethical to conduct an experiment assigning consenting women to breastfeed or formula-feed their infants, because the benefits of breastfeeding are so well-established. But critics question the quality of that evidence, pointing to lack of basis for causal inferences and to the potential for selection bias and confounding to explain correlational effects. Meanwhile, the evidence this article reviewed on preventable harm to neonates associated with breastfeeding insufficiencies suggests that it would also be unethical to conduct an experiment assigning neonates to exclusive breastfeeding within the current paradigm versus an alternative paradigm that returns to prelacteal feeding. These competing claims threaten to create a sort of stagnant King Solomon dilemma in which both ”mothers” refuse to cut the child, leaving the truth contested. 

If this dilemma is valid and irresolvable, scientific institutions charged with interpreting the available evidence should say so and explain what that means for research ethics. Should exclusive breastfeeding versus prelacteal feeding paradigms be compared head-to-head anyway, with adequate informed consent and safeguards, for the betterment of society? Should exclusive formula- versus breastfeeding be similarly compared? Or is the evidence on common and preventable harm to neonates already sufficient to justify a paradigm shift swinging the pendulum back to prelacteal feeding as the norm? If practitioners and researchers should then adhere to new standards for minimizing neonatal starvation/deprivation and later infant under-nutrition periods, what should those standards be? 

In the context of these questions, Congressional requestors might task GAO with evaluating whether recent federally funded research appears to have violated federal law protecting human subjects, constituting fraud (e.g., by concealing information about risks in informed consent protocols that were required to communicate that information), waste, and/or abuse. This would help illustrate bright lines that future science should not cross. They might also task NAM with articulating basic principles and suggestions for more ethically and methodologically rigorous future research on early infant feeding. 

The case for such research proceeding in spite of ethical concerns is pragmatic: There is such a marked disconnect between the evidence on common and preventable harm to neonates from breastfeeding insufficiencies, and current consensus promoting exclusive breastfeeding, that a trial comparing current practice to an alternative paradigm might be necessary to produce the sort of additional, gold-standard evidence enabling causal inferences that may be required to generate broad acceptance of the indicated paradigm shift in early infant feeding. As long as it looks as though healthier children were breastfed and mothers who breastfed are healthier - although this could be for many possible reasons other than straightforward causality, e.g., because healthier mothers and babies are more likely to have breastfeeding success, and / or babies who suffer longer starvation periods due to breastfeeding insufficiencies are more likely to both become formula-fed and to have worse outcomes - medical and public health practitioners are likely to persist in citing correlational evidence as if it were proof of causation, and recommending exclusive breastfeeding for its purported (possible but uncertain) benefits while ignoring its (similarly possible but uncertain) risks. 

A large, well-designed, multi-country trial comparing exclusive breastfeeding versus prelacteal feeding paradigms, and /or exclusive breastfeeding versus formula-feeding groups, could generate novel data including on the effects of relevant post-randomization events such as delayed and insufficient milk, providing vital new insights into the possible roles of neonatal starvation and deprivation in driving outcomes previously attributed to infant feeding mode. Researchers should carefully consider whether and how to block on the most important possible maternal and infant confounds in an attempt to approximately equalize mother-infant dyads with more and less successful breastfeeding across study groups. Potential maternal confounds include primaparity, exhaustion, BMI / metabolic conditions, health status more generally, socio-economic status, and breast abnormalities (such as minimal development during adolescence and/or pregnancy, and inverted nipples); infant confounds include gender, race, and prematurity. Primaparity and prematurity seem most predictive of relevant outcomes of breastfeeding insufficiencies and jaundice vulnerability, respectively. Approximating equalization in this way tends to decrease statistical power while increasing model precision, and would thus make the most sense with a relatively large and diverse sample; but some criticize this approach for its potential to introduce selection bias, recommending simple over stratified randomization for sample sizes over 200 [181, 182]. Thus, simple randomization across diverse trial contexts might best balance the need to generate accurate and credible estimates with the need to measure racial and other subgroup effects. 

This sort of trial has the potential to enable more accurate and credible analyses than ever before on breastfeeding’s effects on child and maternal health, which may include risks and benefits on both sides. This knowledge is of great public health importance. But the ethical implications of the sort of research that might create it are deeply fraught, and require advanced institutional oversight that seems to have been lacking in the field before. Whether a trial like this is conducted or not, available evidence on fully preventable harm to previously healthy neonates from breastfeeding insufficiencies supports greater protections for this particularly vulnerable population in research and practice. 

Moreover, there is ample precedent for evidence-based policy shifts without randomized controlled trials in the history of medicine. For example, when Hungarian physician Ignaz Semmelweis had sufficient theoretical and empirical basis to suspect that doctor handwashing might substantially reduce maternal mortality, in 1847 he implemented the policy that made sense to prevent harm (doctor handwashing), and then analyzed how the change affected maternal deaths after the fact (substantial death reduction) [183]. Germ theory was not yet fully developed or widely accepted; the evidence base for the change had been limited, but sufficient to apply the preventive principle to act to avoid possible risk when the stakes are high. The resulting proof was persuasive enough to change practice eventually. But that change did not come fast enough to prevent Semmelweis from suffering tremendous social and professional harm as colleagues - who his research suggested were responsible for large numbers of preventable deaths - opposed him. When he did not shut up, they ultimately had him committed to an insane asylum where guards beat him and he died, probably from a gangrenous wound sustained during the beating. 

Shifting back from exclusive breastfeeding to an updated prelacteal feeding paradigm presents an analogous case in which the evidence supports immediate policy change to prevent harm, but institutions and experts have incentives to resist the indicated change. Except in this case, there is apparently no Semmelweis in the field working head-on against resistance to the indicated paradigm shift, in the name of science and preventing harm to neonates. And in this case, perverse incentives other than healthcare professionals’ own cognitive dissonance and motivated reasoning (e.g., to believe that they help people rather than harming them) may amplify that resistance: Hospital systems stand to make substantially more money from the risky current paradigm than they would from the safer alternate. 

Under the current system, hospitals profit from insurance claims for rehospitalized newborns who receive in-patient treatment for complications of insufficient milk. Recommending supplementation with sufficient milk guided by infant hunger cues, rather than discouraging such supplementation, would prevent the majority of these hospitalizations. This would frequently decrease hospital revenue, displeasing administrators. 

That is what happened recently at the Isala Hospital in Zwolle, the Netherlands. There, Austie et al [184] replicated earlier results from Harris et al [185] and Hegarty et al [186] showing oral dextrose or glucose reduces hypoglycemia. They reduced the need for IV glucose by half. That cost the hospital nearly 60,000 euros annually. It likely would have cost even more to simply instruct parents to feed neonates formula while establishing safe breastfeeding instead, potentially preventing more medical interventions - oral dextrose/glucose, relevant measurements before and after, and associated consultations - from becoming necessary. 

To address the possibility that hospitals respond to such perverse incentives by maintaining protocols that pose demonstrated risks of preventable harm to newborns, future research might experiment with policy interventions changing those incentives. For instance, health insurance companies might issue a notice that they will refuse to reimburse hospitals for neonatal readmissions within 30 days of birth for fully preventable complications relating to insufficient milk intake in breastfed neonates, such as jaundice, hypoglycemia, and hypernatremia. State or federal legislators might even mandate such a shift. A state-level policy experiment would have the added benefit of generating data that could be compared with data from other states.

## Conclusions

Since the 1970s, evidence has accumulated in the medical literature of common and fully preventable harm to neonates resulting from breastfeeding insufficiencies under the new, historically anomalous exclusive breastfeeding paradigm of early infant feeding. Proponents of this new paradigm tended to lack generations of direct knowledge transmission about safe breastfeeding. They misconceived their intervention as natural and thus safe, and so failed (in good faith) to collect safety data that would have identified these risks. Previous advanced civilizations recognized the common problem of breastfeeding insufficiencies and solved it with safety infrastructure: a well-organized wetnursing profession, shared nursing practices, and prelacteal feeding norms. Modern societies accidentally destroyed that infrastructure, and cannot now easily rebuild it due to contemporary challenges including HIV transmission risk. Early, adequate, and often formula supplementation is the best infrastructural solution to the common breastfeeding insufficiencies problem in the contemporary context. Evidence is insufficient to establish that this alternate paradigm of early infant feeding would necessarily decrease breastfeeding rates, that decreasing exclusive breastfeeding rates would do more harm than starving some neonates as the current paradigm does, or that such decreases would cause harm at all. 

Current early infant feeding guidelines conflict with available evidence on possible risks of breastfeeding insufficiencies. Early insufficient nutrition/hydration has possible long-term effects including neurodevelopmental consequences such as attention deficit hyperactivity disorder, autism, cerebral palsy, cognitive and developmental delay, epilepsy, hearing impairment, kernicterus, language disorder, mood disorders, lower IQ, and specific learning disorder. Responding to this mounting evidence of possible harm, recent medical reform efforts have tended to focus on using more measurement, medical intervention, and technologies within the exclusive breastfeeding paradigm instead of proposing the indicated paradigm shift. But the science supports a simpler solution: application of the precautionary principle to feed hungry neonates the most appropriate available supplemental milk to avoid preventable harm including death and permanent disability. Bringing medical research and practice, public health policy, and societal understandings of safe breastfeeding in line with the scientific evidence presents challenges that likely require political institutional solutions to bring both democratic and expert oversight to bear on medicine and health policy in the public interest. 
